# Children of Senegal River Basin show the highest prevalence of *Blastocystis* sp. ever observed worldwide

**DOI:** 10.1186/1471-2334-14-164

**Published:** 2014-03-25

**Authors:** Dima El Safadi, Lobna Gaayeb, Dionigia Meloni, Amandine Cian, Philippe Poirier, Ivan Wawrzyniak, Frédéric Delbac, Fouad Dabboussi, Laurence Delhaes, Modou Seck, Monzer Hamze, Gilles Riveau, Eric Viscogliosi

**Affiliations:** 1 Institut Pasteur de Lille, Centre d’Infection et d’Immunité de Lille (CIIL), Inserm U1019, CNRS UMR 8204, Université Lille Nord de France, 1 rue du Professeur Calmette, BP 245, 59019 Lille cedex, France; 2 Centre AZM pour la Recherche en Biotechnologie et ses Applications, Laboratoire Microbiologie, Santé et Environnement, Université Libanaise, Tripoli, Lebanon; 3 Biomedical Research Center Espoir pour la Santé (CRB-EPLS), Saint-Louis, Senegal; 4 Microbe Division/Japan Collection of Microorganisms (JCM), RIKEN BioResource Center, Tsukuba, Japan; 5 Laboratoire Microorganismes: Génome et Environnement, CNRS UMR 6023, Université Blaise Pascal de Clermont-Ferrand, Aubière, France

**Keywords:** *Blastocystis* sp, Intestinal parasite, Molecular epidemiology, Pathogenicity, PCR, Subtyping, Transmission, Zoonosis

## Abstract

**Background:**

*Blastocystis* sp. is currently the most common intestinal protist found in human feces and considered an emerging parasite with a worldwide distribution. Because of its potential impact in public health, we reinforced the picture of *Blastocystis* sp. prevalence and molecular subtype distribution in Africa by performing the first survey of this parasite in Senegal.

**Methods:**

Stool samples from 93 symptomatic presenting with various gastrointestinal disorders or asymptomatic children living in three villages of the Senegal River Basin were tested for the presence of *Blastocystis* sp. by non-quantitative and quantitative PCR using primer pairs targeting the SSU rDNA gene. Positive samples were subtyped to investigate the frequency of *Blastocystis* sp. subtypes in our cohort and the distribution of subtypes in the symptomatic and asymptomatic groups of children.

**Results:**

By the use of molecular tools, all 93 samples were found to be positive for *Blastocystis* sp. indicating a striking parasite prevalence of 100%. Mixed infections by two or three subtypes were identified in eight individuals. Among a total of 103 subtyped isolates, subtype 3 was most abundant (49.5%) followed by subtype 1 (28.2%), subtype 2 (20.4%) and subtype 4 (1.9%). Subtype 3 was dominant in the symptomatic group while subtypes 1 and 2 were detected with equal frequency in both symptomatic and asymptomatic groups. The distribution of subtypes was compared with those available in other African countries and worldwide. Comparison confirmed that subtype 4 is much less frequently detected or absent in Africa while it is commonly found in Europe. Potential sources of *Blastocystis* sp. infection including human-to-human, zoonotic, and waterborne transmissions were also discussed.

**Conclusions:**

The prevalence of *Blastocystis* sp. in our Senegalese population was the highest prevalence ever recovered worldwide for this parasite by reaching 100%. All cases were caused by subtypes 1, 2, 3 and 4 with a predominance of subtype 3. More than half of the children infected by *Blastocystis* sp. presented various gastrointestinal disorders. Such high prevalence of blastocystosis in developing countries makes its control a real challenge for public health authorities.

## Background

*Blastocystis* sp. is an anaerobic protist that inhabits the gastrointestinal tract of humans and many groups of animals [1-4]. This cosmopolitan enteric parasite with a worldwide distribution [5] is often identified as the most common unicellular eukaryote reported in human fecal samples [1]. Indeed, its prevalence may exceed 50% in developing countries [6,7] and reach 20% in industrialized countries [8,9]. These differences may be explained by poor sanitary conditions, close animal contact, and consumption of contaminated food or water [10-12]. Such prevalence also suggests large-scale human-to-human [13], waterborne, and zoonotic transmissions for *Blastocystis* sp. [14]. Numerous studies have demonstrated the resistance of cysts of the parasite in feces and environmental sources [15] highlighting the fecal-oral route as the main mode of transmission of *Blastocystis* sp. Moreover, a higher risk of infection has been identified in food and animal handlers, providing conclusive evidence on the transmission of the parasite between humans and animals [16,17].

At the morphological level, *Blastocystis* sp. isolates from humans and animals have been reported to be roughly indistinguishable [1]. However, extensive genetic diversity among *Blastocystis* sp. isolates has been reported based on the comparison of the small-subunit (SSU) rDNA gene sequence [18] leading to the classification of *Blastocystis* sp. isolates into subtypes (STs) [19]. To date, according to recent epidemiological surveys in human and animal populations, 17 STs have been identified [2] but it is highly likely that other STs remain yet to be uncovered. Among the 17 STs, nine of them have been detected in humans with a varying prevalence [5]. Indeed, only four of them are frequent (ST1, ST2, ST3 and ST4) and represent around 90% of the subtyped isolates. A majority of human infections with *Blastocystis* sp. is attributable to ST3 but the distribution of the four predominant STs may vary among the areas studied. Thereby, ST4 is almost as common as ST1 and ST3 in some European countries but seems rare or completely absent in Africa, Asia, and America [5,20]. The other STs that are common in animal hosts i.e. ST5 to ST9, are rarely found in humans and are most likely the result of zoonotic transmission.

As *Blastocystis* sp. can be found in both symptomatic and asymptomatic patients its clinical relevance remained uncertain [1]. However, a converging bundle of recent *in vivo*, *in vitro* and genomic data strongly suggests that this microorganism may be pathogen [21-24] and allows proposing a model for pathogenesis of this parasite [25] particularly involving virulence factors as cysteine proteases [26,27]. Therefore, *Blastocystis* sp. should be associated with a variety of non-specific gastrointestinal disorders including diarrhea, abdominal pain, nausea and vomiting [1,22] and also suspected to be linked to irritable bowel syndrome (IBS) [25,28] and chronic or acute urticarial lesions [29,30]. In addition, *Blastocystis* sp. has increasingly been implicated in diarrheal illness in immunocompromised individuals including HIV/AIDS and cancer patients and transplant recipients [31,32].

It has long been suggested that the pathogenesis of *Blastocystis* sp. may be dependent upon ST but recent epidemiological data remain contradictory [8,29,33-36]. Nevertheless, the likely pathogenic power of *Blastocystis* sp. coupled to its high prevalence in the human population raise crucial questions about its current burden in public health. Consequently, information on the prevalence of the parasite and the distribution of STs are starting to emerge in still poorly studied geographic areas as Africa. To date the available data concern only few African countries of the North and West such as Egypt [21,37,38], Libya, Nigeria, and Liberia [5] and one Eastern African country, Tanzania [39]. Therefore, the aim of the present study was to reinforce the picture of *Blastocystis* sp. prevalence and ST distribution in Africa by performing the first survey of this parasite in Senegal. In this epidemiological study, we described the prevalence of *Blastocystis* sp. infection and ST distribution in a cohort of Senegalese children living in 3 rural villages in close contact with animals and with poor or no access to clean water and sanitation. The distribution of *Blastocystis* sp. STs in both asymptomatic and symptomatic individuals was investigated together with potential risk factors for transmission of the parasite.

## Methods

### Cohort and collection of samples

This work has been carried out as part of the "SchistoVAN" project (clinicaltrials.gov ID NCT01553552), sponsored by the Biomedical Research Centre EPLS (Saint-Louis, Senegal) ( http://www.espoir-sante.org). The present study was approved by the National Ethics Committee of the Ministry of Health of Senegal (September 2011; protocol number SEN11/43, clinicaltrials.gov: NCT01553552). Oral and written informed consents were obtained from the parents or the legal guardians of the children in accordance with the Code of Ethics of the World Medical association (Declaration of Helsinki). Children enrolled in the present study formed a sub-cohort of the "AnoPalAnoVac" project (clinicaltrials.gov ID NCT01545115) and were recruited on the basis of their age (6 to 10 years of age in October 2011) through village nurses, healthcare workers in the community and school directors. Date of birth was ascertained from vaccination cards or school register. A standardized questionnaire was completed for each child (see Additional file 1). In particular, children were asked about each gastrointestinal symptom individually including abdominal pain, diarrhea, and vomiting. This cross-sectional study was conducted in October 2011, in 3 villages of the Podor district located in northern Senegal: Agniam Towguel (16°32′N-14°48′W; total population (TP): 989; several temporary ponds, traditional housing, irrigated crops), Fanaye Diery (16°32′N-15°13′W; TP: 6781; animal husbandry, irrigated crops, some urbanized habitat), and Niandane (16°35′N-14°59′W; TP: 5100; rice and banana farming, irrigated crops, some urbanized habitat, no access to running water). In this region, the climate is Sahelian, with annual rainfall between July and September. Mean temperature ranges between 20°C and 30°C during the cool season (November to February) and between 25°C and 38°C during the warm season (March to October). In this study, stool samples were collected from 93 symptomatic and asymptomatic children (Agniam = 17; Fanaye = 38; Niandane = 38) presenting or not gastrointestinal disorders. These boys (n = 46) and girls (n = 47) were aged 6 to 10 years (median age 8.4; quartiles: 6.6; 9.5) (Table 1). Children presented no symptoms of severe illness and/or fever and were subject to a questionnaire that was designed to collect clinical data especially regarding recent diarrhea, vomiting, and abdominal pain. For storage and transport at -20°C to Lille, stool samples were added in Stool Transport and Recovery (S.T.A.R.) buffer (Roche) in a ratio of 1:3 according to the manufacturer’s recommendations then homogenized by shaking.

**Table 1:** **Clinical data and *Blastocystis *** **sp. STs among symptomatic and asymptomatic patients in Senegal**

**Patients**	**Sex/age**	**Symptoms**	***Blastocystis *** **sp. ST by non-qPCR**^**a**^	**Nucleotide differences**^**b**^	***Blastocystis *** **sp. ST by qPCR**	**Accession no.**
DSS1	M/6.2	Abdominal pain			1	KF848509
DSS2	F/5.9	Abdominal pain, diarrhea	3 (Sc)	0		KF848510
DSS3	M/6.8		3 (Sc)			KF848511
DSS4	F/6.1	Abdominal pain, vomiting	1 (Sc)			KF848512
DSS5	M/8.3				3	KF848513
DSS6	M/7.3	Abdominal pain, diarrhea	1 (Sc)			KF848514
DSS7	F/5.9				3	KF848515
DSS8	M/6.8	Abdominal pain, vomiting	1 (Sc)			KF848516
DSS9	F/9.2	Abdominal pain			3	KF848517
DSS10	M/9.7	Vomiting	3 (Sc)	0		KF848518
DSS11	F/10	Diarrhea, vomiting	3 (Sc)			KF848519
DSS12	M/8.8	Abdominal pain, vomiting			3	KF848520
DSS13	F/8.6	Abdominal pain	1 (Sc)			KF848521
DSS14	M/7		2 (Sc)	0		KF848522
DSS15	F/7.7		2 (Sc)			KF848523
DSS16	M/6.9				3	KF848524
DSS17	F/7.7				3	KF848525
DSS18	F/6.1		3 (Sc)	0 to14		KF848526-7
DSS19	M/8.7				2	KF848528
DSS20	M/9.7	Abdominal pain	2 (Sc)			KF848529
DSS21	F/6	Abdominal pain	1 (Sc)			KF848530
DSS22	M/10.1	Abdominal pain	3 (Sc)			KF848531
DSS23	M/7.7		Mixed 2 and 3 (P)	0		KF848532-3
DSS24	F/6.4		1 (Sc)			KF848534
DSS25	F/9.6	Abdominal pain	Mixed 3 and 4 (Sc)	0 to 4		KF848535-7
DSS26	F/6.5	Abdominal pain	Mixed 1, 2 and 3(Sc)			KF848538-40
DSS27	M/7.2		3 (Sc)			KF848541
DSS28	M/8		3 (Sc)			KF848542
DSS29	F/7.3				2	KF848543
DSS30	M/5.8	Abdominal pain			1	KF848544
DSS31	F/8.6	Abdominal pain	2 (Sc)			KF848545
DSS32	M/6.6		3 (Sc)			KF848546
DSS33	M/7.7	Abdominal pain	Mixed 2 and 3 (Sc)	0		KF848547-8
DSS34	M/9.5	Vomiting	3 (Sc)			KF848549
DSS35	M/7.7	Abdominal pain	3 (Sc)	0		KF848550
DSS36	F/5.9	Abdominal pain	3 (Sc)			KF848551
DSS37	M/7.4		2 (Sc)	0		KF848552
DSS38	F/9.7	Abdominal pain, diarrhea, vomiting	1 (Sc)	0		KF848553
DSS39	F/9.8	Abdominal pain			3	KF848554
DSS40	F/9.9	Abdominal pain, diarrhea, vomiting	2 (Sc)			KF848555
DSS41	F/9.7	Vomiting	3 (Sc)	0		KF848556
DSS42	F/10.2	Abdominal pain, diarrhea	1 (Sc)			KF848557
DSS43	M/9.3		2 (Sc)			KF848558
DSS44	M/10	Abdominal pain	3 (Sc)			KF848559
DSS45	F/8.9	Abdominal pain	3 (Sc)	0 to 14		KF848560-1
DSS46	F/10.2	Abdominal pain			3	KF848562
DSS47	F/10		3 (Sc)	0		KF848563
DSS48	M/9.8		3 (Sc)	0		KF848564
DSS49	F/10		2 (Sc)			KF848565
DSS50	F/10.2				1	KF848566
DSS51	M/9.4		1 (Sc)			KF848567
DSS52	M/9	Abdominal pain	1 (Sc)			KF848568
DSS53	F/8.1		1 (Sc)			KF848569
DSS54	M/8.5		Mixed 1 and 2 (Sc)	0		KF848570-1
DSS55	M/9.1	Abdominal pain	3 (Sc)			KF848572
DSS56	F/8.6		1 (Sc)			KF848573
DSS57	F/9.7	Abdominal pain	2 (Sc)			KF848574
DSS58	M/9.3	Abdominal pain	2 (Sc)			KF848575
DSS59	M/8.9	Abdominal pain	3 (Sc)			KF848576
DSS60	F/8.8		3 (Sc)			KF848577
DSS61	M/9.9				1	KF848578
DSS62	F/9.5	Abdominal pain			1	KF848579
DSS63	M/8.5	Abdominal pain	1 (Sc)			KF848580
DSS64	M/6.2	Abdominal pain	3 (Sc)			KF848581
DSS65	M/9.8		1 (Sc)			KF848582
DSS66	F/9.9	Abdominal pain	2 (Sc)			KF848583
DSS67	F/6.1	Abdominal pain	Mixed 1 and 4 (Sc)	0		KF848584-5
DSS68	F/6.1		1 (Sc)	0		KF848586
DSS69	F/6.8				1	KF848587
DSS70	F/7.8	Abdominal pain	3 (Sc)			KF848588
DSS71	F/6.7	Abdominal pain			3	KF848589
DSS72	M/6	Abdominal pain, vomiting	3 (Sc)			KF848590
DSS73	F/7	Abdominal pain	3 (Sc)	0		KF848591
DSS74	M/8.5				3	KF848592
DSS75	M/5.9	Abdominal pain			3	KF848593
DSS76	M/8.1	Abdominal pain			1	KF848594
DSS77	M/6.6	Abdominal pain	3 (Sc)	2 to 12		KF848595-7
DSS78	M/6.9	Abdominal pain			2	KF848598
DSS79	M/8.4		3 (Sc)	0 to 11		KF848599-600
DSS80	M/6.1				3	KF848601
DSS81	F/5.9	Abdominal pain	Mixed 2 and 3 (Sc)	0		KF848602-3
DSS82	F/9.8	Abdominal pain	3 (Sc)	0		KF848604
DSS83	M/6.4				1	KF848605
DSS84	F/9.2		2 (Sc)			KF848606
DSS85	F/6.8	Abdominal pain, vomiting	3 (P)	3 to 5		KF848607-9
DSS86	F/6.6	Abdominal pain	Mixed 1, 2 and 3 (P)			KF848610-2
DSS87	M/9.9	Abdominal pain	3 (P)	0		KF848613
DSS88	M/9.5		1 (P)	0 to 1		KF848614-5
DSS89	F/9.1	Diarrhea	3 (P)	0		KF848616
DSS90	F/9.5		3 (P)	0 to 1		KF848617-8
DSS91	F/9.4		1 (P)	0 to 3		KF848619-20
DSS92	F/6.1	Abdominal pain, diarrhea	3 (P)	0 to 3		KF848621-2
DSS93	M/6		3 (P)	0 to 1		KF848623-4

^a^According to the standard terminology including 17 STs [2]; (Sc) and (P): non qPCRs using the primer pair described by Scicluna et al. [40] and Poirier et al. [20], respectively.

^b^Determined in the common region of clones belonging to the same ST and sequenced for each sample.

### DNA extraction and molecular subtyping of *Blastocystis* sp. isolates

Samples stored in S.T.A.R. buffer were stirred and then centrifuged for 1 min at 1,000 g. Total genomic DNA was extracted from 200 μl of the cleared stool supernatant using the QiaAMP DNA Stool Mini Kit (Qiagen, Hilden, Germany) according to the manufacturer’s instructions. For each sample, 5 μl of extracted DNA was submitted to non-quantitative Polymerase Chain Reactions (non-qPCRs) as previously described using two independent pairs of *Blastocystis* sp.-specific primers designed by Scicluna et al. [40] and Poirier et al. [20], both targeting the small subunit (SSU) rDNA coding region. The respective 600 bp- and 320 bp-amplified domains have been shown to contain sufficient information for accurate subtyping of *Blastocystis* sp. isolates [20,40]. Non-qPCR amplifications were carried out in 50 μl according to standard conditions for Platinum *Taq* High-Fidelity DNA polymerase (Invitrogen). For each DNA sample, the non-qPCR product with the highest intensity on agarose gel was purified and sequenced. Direct sequencing of few non-qPCR products generated mixed signals that could reflect infections by different STs or was of poor quality probably due to a too small amount of parasite DNA matrix. Because of the difficulty in interpreting the corresponding DNA sequencing chromatograms, same samples were re-analyzed by non-qPCRs and positive bands of the expected sizes were purified and cloned as previously described [41]. Three positive clones containing inserts were arbitrarily selected for each sample and sequenced on both strands. The DNA samples negative by non-qPCRs were subsequently amplified using the real time qPCR assay developed by Poirier et al. [20]. Due to the high sensitivity of this detection method, various controls were performed to prevent artifacts related to contamination through different sources: DNA extraction controls (isolation of DNAs without stool and from a *Blastocystis* sp.-negative stool) subsequently used in qPCR assays and negative (DNA matrix replaced by water) and positive (DNA obtained from a *Blastocystis* sp. ST4 culture) qPCR controls. The expected 320 bp-amplified variable region of the SSU rRNA gene was directly sequenced for subtyping. The SSU rRNA gene sequences obtained in this study have been deposited in GenBank under accession numbers KF848509 to KF848624. Obtained sequences were compared with all *Blastocystis* sp. SSU rRNA gene sequences available from the National Centre for Biotechnology Information (NCBI) using the BLAST program. STs were identified by determining the exact match or closest similarity against all known *Blastocystis* sp. STs according to the updated classification by Alfellani et al. [2].

## Results and discussion

### Screening for *Blastocystis* sp. in a cohort of Senegalese children using molecular methods

In this study, fecal samples were collected from 93 children, 46 boys and 47 girls, aged 6 to 10 years and living in 3 villages situated in the north of Senegal. This cohort consisted of two groups: one symptomatic composed of 54 children presenting variously with abdominal pain, vomiting, and diarrhea and one asymptomatic including 39 other children (Table 1). All 93 samples were screened for *Blastocystis* sp. by non-qPCR and qPCR methods using specific primer pairs amplifying distinct domains of the SSU rDNA coding region [20,40]. Non-qPCR products were directly sequenced or subsequently cloned whether the resulting sequence chromatogram showed double or poor quality signals. In the latter case that involved 32 samples, three positive clones containing inserts were arbitrarily selected and sequenced. Overall, 71 samples were identified as positive for *Blastocystis* sp. by non-qPCR methods. The remaining 22 samples negative by non-qPCR nevertheless proved to be positive for the parasite by qPCR. Thereby, by means of these different molecular approaches, *Blastocystis* sp. was recognized in 100% of stool samples of this Senegalese population. To our knowledge this represents the highest prevalence ever recovered worldwide for this parasite.

In almost all molecular epidemiological studies conducted to date, only one nucleic acid-based diagnostics methodology was used to identify and subtype *Blastocystis* sp. isolates in stool samples. Briefly, PCR screening of fecal samples can be achieved using ST-specific sequence-tagged-site (STS) primers or genus-specific primers with subsequent sequencing for ST identification. Several regions in the SSU rDNA gene have been proposed by different authors for sequencing [24] but the "barcode" region designed by Scicluna et al. [40] has been extensively used as in the present study. Advantages and limitations of these two PCR approaches were recently largely discussed [24,42] and a comparative study using both techniques recommended barcoding as the relevant method for *Blastocystis* sp. diagnosis and subtyping [43]. However, as confirmed from our data, direct sequencing of non-qPCR products may be unsuccessful in the case of mixed infections with more than one *Blastocystis* sp. ST in the same sample. Therefore subtyping of isolates in mixed infections requires cloning of the non-qPCR product and sequencing of several clones as previously suggested [44]. In addition, about 24% of the samples have not been diagnosed as positive for *Blastocystis* sp. by non-qPCR methods in our study. Hence, at this stage of our screening, the prevalence of the parasite was 76%, which greatly underestimated its true prevalence as determined in a second step by qPCR (100%). Consequently, our study confirmed the impact of detection methods to ascertain the actual prevalence of *Blastocystis* sp. in human populations and the interest and higher sensitivity of qPCR assays in comparison to non-qPCR methods for epidemiological surveys.

Although the prevalence of *Blastocystis* sp. never reached 100% in any cohort studied so far, it was described as very high in some developing countries specially, in children. By using non-molecular standard methods, the prevalence of the parasite already exceeded 40% in communities in Venezuela [45], Peru [46], Colombia [7], and in school children or orphanage in Indonesia [6], Thailand [47], and Lebanon (El Safadi unpubl.). Moreover, the prevalence of *Blastocystis* sp. was around 60% in immigrants in the city of Naples (southern Italy) who came for a large majority from northern and western African countries [48]. In Libya, the prevalence of the parasite has been previously reported close to 30% [49]. In Senegal, although the prevalence of *Blastocystis* sp. had never been determined before, intestinal parasites (protozoa and helminths) represent for a long time a major public health problem. Indeed, the global prevalence of intestinal parasites in children ranged from 32% to 84% depending on the study areas [50-53]. Such impact of intestinal parasites previously reported in the Senegalese population is clearly in accordance with the pattern found for *Blastocystis* sp. in the current study.

### Distribution of *Blastocystis* sp. STs in the Senegalese population and comparison with other African countries

In all the children, the expected SSU rDNA domains targeted by non-qPCR and qPCR methods were amplified and successfully sequenced. Each of the new SSU rDNA gene sequences obtained in this study showed very high similarity (98% to 100%) to homologous sequences of the other *Blastocystis* sp. isolates reported so far. The comparison with representative sequences of all known *Blastocystis* sp. STs allowed the direct subtyping of the new isolates (Table 1). As stated above, for 32 of the 93 samples, cloning of the non-qPCR product was necessary for subtyping and 3 positive clones were arbitrarily selected and sequenced. For 14 of these 32 samples, the three sequenced clones were identical (Table 1). For 10 other samples presenting infection by a single ST, clones showed 1 to 14 nucleotide differences between them. As previously suggested [40,41], these nucleotide differences may reflect either coinfection with two isolates of the same ST or sequence variations between SSU rDNA gene copies within the same isolate [23]. Interestingly, the higher number of nucleotide difference between the clones (11 to 14) was observed in children infected by ST3. Recently, by analyzing and comparing respectively SSU rDNA sequences or data from multilocus sequence typing, Meloni et al. [44] and Stensvold et al. [54] revealed substantial intra-ST diversity in ST3 and more limited intra-ST2 and intra-ST4 variations. In either case, we have considered in the following analysis of our data that two different clones of the same ST identified in the same patient derived from the same isolate of *Blastocystis* sp. For the last 8 samples, mixed infections with two (6 samples) or three STs (2 samples) were identified (Table 2). The majority of the subtyped samples included in the present study (85/93) represented single infections. With the addition of 8 mixed infections consisting of two or three different isolates, we analyzed a total of 103 isolates. As summarized in Table 3, ST3 was the most common *Blastocystis* sp. ST in our Senegalese population (49.5%) followed by ST1 (28.2%), ST2 (20.4%) and ST4 (1.9%). The distribution of STs was almost similar between the three villages with a majority of infections attributable to ST3. The only significant difference was that ST1 followed at position 2 in Niandiane and Agniam Towguel and at position 3 in Fanaye Diery.

**Table 2:** **Single and mixed ST infections with *Blastocystis *** **sp. identified in the Senegalese cohort**

**Villages**	***Blastocystis *** **sp. STs**								**Total no.**
	**ST1**	**ST2**	**ST3**	**ST1/ST2**	**ST1/ST4**	**ST2/ST3**	**ST3/ST4**	**ST1/ST2/ST3**	
**Agniam Towguel**	5	2	9	0	0	0	0	1	17
**Niandane**	13	5	17	1	1	1	0	0	38
**Fanaye Diery**	7	8	19	0	0	2	1	1	38
**Total no.**	25	15	45	1	1	3	1	2	93

**Table 3:** ***Blastocystis *** **sp. ST distribution among Senegalese villages**

**Villages**	***Blastocystis *** **sp. STs (%)**				**Total no.**
	**ST1**	**ST2**	**ST3**	**ST4**	
Agniam Towguel	6	3	10	0	19
Niandane	15	7	18	1	41
Fanaye Diery	8	11	23	1	43
Total no. (%)	29 (28.1)	21 (20.3)	51(49.5)	2 (1.9)	103

**Figure 1:**
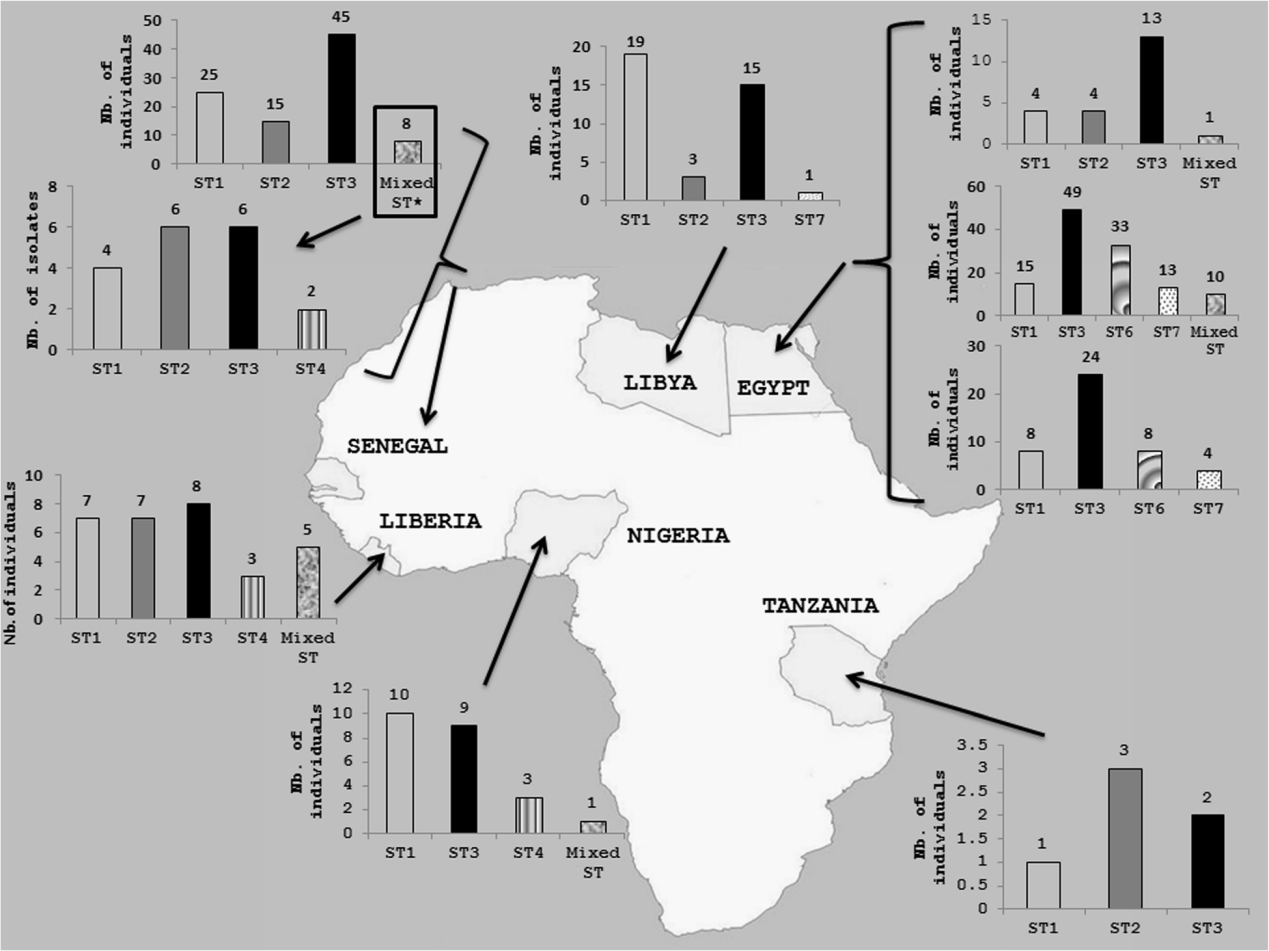
**Distribution of *Blastocystis *sp. STs across African countries.** In the case of Senegal, distribution of STs in mixed infections indicated by an asterisk in the histogram was detailed.

The distribution of *Blastocystis* sp. STs in Senegal is quite similar to that observed in a majority of countries all over the world with a predominance of ST3 followed by ST1 then ST2 or ST4 [5]. If we focused on the studies previously conducted in Africa, the only available reports concerned the ST distribution in Egypt [21,37,38], Libya, Nigeria, Liberia [2], and Tanzania [39], the latter survey only including 6 isolates (Figure 1). Globally, ST1, ST2, and ST3 represent around 82% of all 369 African isolates subtyped so far including those of the present study. ST3 is highly prevalent in African countries with an average of 46% of the 369 isolates. Consequently, because ST3 is rarely found in animals, a large-scale human-to-human transmission might likely explain the predominance of this ST in African countries as also in the rest of the world. Three studies conducted in different cities in Egypt and that in Liberia have identified ST3 as the predominant ST, as was true for our study population. However ST1 was detected with the highest frequency in Libya and Nigeria, and ST2 in Tanzania. Since ST1 and ST2 are frequently found in various animal groups including livestock [2,4,55], it can be hypothesized that a not yet evaluated proportion of human infections by both STs are of zoonotic origin. Interestingly ST4 was not detected in the studies reported in Egypt, Libya and Tanzania and its prevalence is quite low in Senegal (1.9%). Although ST4 was relatively frequent in Liberia (12%) and Nigeria (14%), these data confirmed that ST4 is much less frequently detected or absent in Africa while it is commonly found in Europe [5,34]. However, the reasons for the heterogeneous geographical distribution of ST4 remain unknown and its reservoir hosts have to be clarified. Additional STs including ST6 and ST7 were commonly found only in two studies carried out in Egypt [21,38]. Indeed, these STs were not detected in Senegal, Liberia, Nigeria, and Tanzania as in a third survey in Egypt [37] and only one isolate belonging to ST7 was identified in Libya. Both STs are usually hosted by birds (avian STs) [56] and rarely found in mammals. This was confirmed in the present study. Given their apparent host specificity, it is highly likely that human infections by avian STs are of zoonotic origin.

The prevalence of mixed ST infections was 8.6% (8/93 samples) in our Senegalese cohort. This percentage is probably underestimated but roughly similar to that calculated (6%) by taking into account subtyped isolates from all epidemiological studies published to date (191 of 3171 samples) and listed by Alfellani et al. [5]. Mixed ST infections were found in all three villages of our study but with different proportions. In Agniam Towguel, only one mixed infection by 3 STs was found (ST1/ST2/ST3) (5.9%) while 4 mixed infections by 2 or 3 STs (10.5%) (ST2/ST3, ST3/ST4 and ST1/ST2/ST3) were recorded in Fanaye Diery. Regarding Niandane, 3 mixed infections by 2 STs (7.9%) were identified (ST1/ST2, ST1/ST4, and ST2/ST3) (Table 2). Mixed infections mostly corresponded to ST1, ST2, and ST3 isolates and were more likely to occur considering the high prevalence of each of these STs in our cohort. Curiously, the only two ST4 isolates of our study were identified in the context of mixed infections and not as part of single infections.

### Distribution of *Blastocystis* sp. STs in symptomatic and asymptomatic groups

According to recent epidemiological reports, links between ST and pathogenicity are still matter of debate [8,29,33-36,41]. To highlight a possible relationship between ST and major intestinal symptoms associated with *Blastocystis* sp. infection (diarrhea, vomiting, and abdominal pain), the ST distribution was evaluated in our cohort of 93 symptomatic or asymptomatic children. Interestingly, the majority of children infected by *Blastocystis* sp. (54/93 or 58%) presented various gastrointestinal disorders as previously observed in a pediatric unit in Turkey [57]. In the following analysis, symptomatic (6) and asymptomatic (2) children presenting mixed infections by two or three STs were removed. Thereby the remaining 48 isolates from symptomatic children and 37 isolates from asymptomatic children were considered (Table 4). The most dominant ST in both groups was ST3, and 63% of the ST3 isolates were found in symptomatic individuals. Predominance or high prevalence of ST3 in symptomatic groups of patients was also shown for instance in France [41], Australia [8], Turkey [57,58], and Iran [35]. Regarding ST1 and ST2 isolates, both STs were detected with equal frequency in the symptomatic and asymptomatic groups of our cohort (Chi square test, p = 0.845). However, ST1 was over-represented in groups of symptomatic individuals in previous epidemiological studies in China [59], Turkey [60], Iran [35], Egypt [21], and Lebanon [36]. Moreover, ST1 was the most prevalent ST in some cohorts presenting with IBS [38,61] and was shown to be pathogenic in rat models of blastocystosis [21]. In contrast, ST2 was not usually overrepresented in groups of symptomatic patients from different countries and Dogruman-Al et al. [57] suggested that ST2 was a non-pathogenic ST. However, in our study, the proportion of ST2 isolates in symptomatic patients was far from negligible. Moreover, all ST2 isolates identified in French and Australian sampling were from symptomatic individuals [8,41]. To complete our observations, it can also be underlined that the only two ST4 isolates found in our study were associated with symptoms but in the context of mixed ST infections (associated to ST1 or ST3). Therefore, the pathogenic potential of ST4 could not be assessed from our epidemiological data. However, a high prevalence of ST4 was recently found in Danish patients presenting with acute diarrhea [33] and Spanish symptomatic individuals [62]. Globally, no association between ST and symptom status was statistically significant from our present data.

**Table 4:** **Distribution of *Blastocystis *** **sp. STs among symptomatic and asymptomatic children groups**

**Group**	***Blastocystis *** **sp. STs (%)**				**Total no.**
	**ST1**	**ST2**	**ST3**	**Mixed infections**	
Symptomatic	13 (24)	7 (13)	28 (52)	6 (11)	54
Asymptomatic	12 (31)	8 (20)	17 (44)	2 (5)	39
**Total no (%)**	**25 (27)**	**15 (16)**	**45 (48)**	**8 (9)**	**93**

### Potential risk factors for transmission of *Blastocystis* sp. within the Senegalese cohort

The fecal-oral route is the main mode of transmission of *Blastocystis* sp. like the other common gastrointestinal parasites [1]. To reach a prevalence of 100% as in the present study, it is reasonable to argue that infection of Senegalese children by the parasite occurred through multiple transmission sources. Although we did not had the opportunity to collect and analyze environmental (mainly water samples) and animal samples, it appears clear that individuals living in small communities in a rural area with modest socio-economic status (average income of 0.5 $ per person per day in these Senegalese villages), poor hygiene sanitation, close contact with domestic animals and livestock, and water supply directly from well and river as in our study greatly increase their risks of infection by *Blastocystis* sp. Indeed, recent studies have revealed a human-to-human transmission coupled with a high prevalence of the parasite in a close community of girls in Thailand [13] as well as in a rural village in Nepal [14] whose description and environment (animals reared in or next to villager dwellings and rivers as water sources of the community) are quite similar to those of Senegalese villages. In this context, zoonotic transmission of *Blastocystis* sp. would be another significant source of infection due to the wide range of animals potentially infected with the parasite and in close contact with humans [2,4,55,56]. However the impact of this mode of transmission through contact with contaminated animal fecal materials coupled with poor hand hygiene of villagers remains difficult to quantify therefore that it involves STs frequently found in humans (ST1 to ST4) as in the present study. Finally, there is a concern that water should be a main source of infection by *Blastocystis* sp. although large-scale waterborne outbreaks involving the parasite have not yet been documented. Ability of *Blastocystis* sp. cysts, the only transmissible stage of this parasite to survive for long periods of time in the environment together with their low size [1,15] represent some factors that favor waterborne transmission of the parasite. In 2006, *Blastocystis* sp. has also been added by the World Health Organization to the list of waterborne parasites [63]. *Blastocystis* sp. was previously reported in drinking water, rivers, tanks, and sewage samples [11,12,14,15,64]. Interestingly, in these latter studies, the same STs were identified in both the water samples and the stools of consumers providing evidence for water transmission. In the Senegalese villages of the present study, river and wells represent the main water sources for drinking and cooking purposes as well as for bathing of the population. Consequently, water sources of this community potentially contaminated with human and animal feces, may largely facilitate transmission of *Blastocystis* sp. and should be considered as serious potential threats to consumers. Further, the analysis of fecal samples from animals living in or near the Senegalese villages and of water samples would help to confirm their epidemiological importance as sources of infection and contamination for inhabitants.

## Conclusions

To our knowledge, this is the first investigation of prevalence and ST distribution of *Blastocystis* sp. in Senegal that, moreover, greatly expands the few data available on molecular epidemiology of this parasite in Africa. In this country, the prevalence of *Blastocystis* sp. in a cohort of children living in a rural area reached a peak of 100% never achieved before. Such high prevalence may reflect different exposure of individuals to animal (zoonosis) and environmental (waterborne) sources of infection together with large-scale human-to-human transmission. This study also raises questions about the real pathogenicity of *Blastocystis* sp. since more than half of children infected by this parasite presented various gastrointestinal symptoms and highlights the socioeconomic impact of blastocystosis in developing countries with low environmental conditions and quality of life. Finally, this epidemiological survey provides necessary information to public health authorities for urgently setting prevention (water quality assessments and primary education of dwellers regarding the modes of transmission) and control programs that should help to reduce the burden of *Blastocystis* sp.

## Abbreviations

ST: Subtype; IBS: Irritable bowel syndrome.

## Competing interests

The authors declare that they have no competing interests.

## Authors’ contributions

LG and MS collected stools and patient data and helped in drafting the manuscript. DES, DM, AC, PP and IW collected and analyzed PCR and sequence data and helped in drafting the manuscript. EV, GR, MH, LD, FDe and FDa conceived, designed and coordinated the study and drafted the manuscript. All authors read and approved the final manuscript.

## Pre-publication history

The pre-publication history for this paper can be accessed here:

http://www.biomedcentral.com/1471-2334/14/164/prepub

## Supplementary Material

Additional file 1 Standardized questionnaire completed for each child in the study. Click here for file

## References

[B1] Tan KS New insights on classification, identification, and clinical relevance of *Blastocystis* spp Clin Microbiol Rev 2008 14 639 665 10.1128/CMR.00022-08 18854485 PMC2570156

[B2] Alfellani MA Taner-Mulla D Jacob AS Imeede CA Yoshikawa H Stensvold CR Clark CG Genetic diversity of *Blastocystis* in livestock and zoo animals Protist 2013 14 497 509 10.1016/j.protis.2013.05.003 23770574

[B3] Alfellani MA Jacob AS Perea NO Krecek RC Taner-Mulla D Verweij JJ Levecke B Tannich E Clark CG Stensvold CR Diversity and distribution of Blastocystis sp. subtypes in non-human primates Parasitology 2013 14 966 971 10.1017/S0031182013000255 23561720

[B4] Roberts T Stark D Harkness J Ellis J Subtype distribution of *Blastocystis* isolates from a variety of animals from South Wales, Australia Vet Parasitol 2013 14 85 89 10.1016/j.vetpar.2013.01.011 23398989

[B5] Alfellani MA Stensvold CR Vidal-Lapiedra A Onuoha ES Fagbenro-Beyioku AF Clark CG Variable geographic distribution of *Blastocystis* subtypes and its potential implications Acta Trop 2013 14 11 18 10.1016/j.actatropica.2012.12.011 23290980

[B6] Pegelow K Gross R Pietrzik K Lukito W Richards AL Fryauff DJ Parasitological and nutritional situation of school children in the Sukaraja district, West Java, Indonesia Southeast Asian J Trop Med Public Health 1997 14 173 190 9322303

[B7] Ramirez JD Sanchez LV Bautista DC Corredor AF Florez AC Stensvold CR *Blastocystis* subtypes detected in humans and animals from Colombia Infect Genet Evol 2013 doi:10.1016/j.meegid.2013.07.020. [Epub ahead of print] 10.1016/j.meegid.2013.07.020 23886615

[B8] Roberts T Stark D Harkness J Ellis J Subtype distribution of *Blastocystis* isolates identified in a Sydney population and pathogenic potential of *Blastocystis* Eur J Clin Microbiol Infect Dis 2013 14 335 343 10.1007/s10096-012-1746-z 22996007

[B9] Bart A Wentink-Bonnema EMS Gilis H Verhaar N Wassenaar CJA van Vugt M Goorhuis A van Gool T Diagnosis and subtype analysis of *Blastocystis* sp. in 442 patients in a hospital setting in the Netherlands BMC Infect Dis 2013 14 389 10.1186/1471-2334-13-389 23972160 PMC3765316

[B10] Li LH Zhou XN Du ZW Wang XZ Wang LB Jiang JY Yoshikawa H Steinmann P Utzinger J Wu Z Chen JX Chen SH Zhang L Molecular epidemiology of human *Blastocystis* in a village in Yunnan province, China Parasitol Int 2007 14 281 286 10.1016/j.parint.2007.06.001 17627869

[B11] Leelayoova S Siripattanapipong S Thathaisong U Naaglor T Taamasri P Piyaraj P Mungthin M Drinking water: a possible source of *Blastocystis* spp. subtype 1 infection in schoolchildren of a rural community in central Thailand Am J Trop Med Hyg 2008 14 401 406 18784233

[B12] Eroglu F Koltas IS Evaluation of the transmission mode of *B. hominis* by using PCR method Parasitol Res 2010 14 841 845 10.1007/s00436-010-1937-4 20544220

[B13] Thathaisong U Siripattanapipong S Mungthin M Pipatsatitpong D Tan-ariya P Naaglor T Leelayoova S Identification of *Blastocystis* subtype 1 variants in the home for girls, Bangkok, Thailand Am J Trop Med Hyg 2013 14 352 358 10.4269/ajtmh.2012.12-0237 23166199 PMC3583329

[B14] Lee LL Chye TT Karmacharya BM Govind SK *Blastocystis* sp.: waterborne zoonotic organism, a possibility? Parasit Vectors 2012 14 130 10.1186/1756-3305-5-130 22741573 PMC3434050

[B15] Suresh K Smith H Tan T Viable *Blastocystis* cysts in Scottish and Malaysian sewage samples Appl Environ Microbiol 2005 14 5619 5620 10.1128/AEM.71.9.5619-5620.2005 16151162 PMC1214661

[B16] Yoshikawa H Wu Z Pandey K Pandey BD Sherchand JB Yanagi T Kanbara H Molecular characterization of *Blastocystis* isolates from children and rhesus monkeys in Kathmandu, Nepal Vet Parasitol 2009 14 295 300 10.1016/j.vetpar.2008.11.029 19136214

[B17] Parkar U Traub RJ Vitali S Elliot A Levecke B Robertson I Geurden T Steele J Drake B Thompson RC Molecular characterization of *Blastocystis* isolates from zoo animals and their animal-keepers Vet Parasitol 2010 14 8 17 10.1016/j.vetpar.2009.12.032 20089360

[B18] Noël C Dufernez F Gerbod D Edgcomb VP Delgado-Viscogliosi P Ho LC Singh M Wintjens R Sogin ML Capron M Pierce R Zenner L Viscogliosi E Molecular phylogenies of *Blastocystis* isolates from different hosts: implications for genetic diversity, identification of species, and zoonosis J Clin Microbiol 2005 14 348 355 10.1128/JCM.43.1.348-355.2005 15634993 PMC540115

[B19] Stensvold CR Suresh GK Tan KS Thompson RC Traub RJ Viscogliosi E Yoshikawa H Clark CG Terminology for *Blastocystis* subtypes–a consensus Trends Parasitol 2007 14 93 96 10.1016/j.pt.2007.01.004 17241816

[B20] Poirier P Wawrzyniak I Albert A El AH Delbac F Livrelli V Development and evaluation of a real-time PCR assay for detection and quantification of *Blastocystis* parasites in human stool samples: prospective study of patients with hematological malignancies J Clin Microbiol 2011 14 975 983 10.1128/JCM.01392-10 21177897 PMC3067686

[B21] Hussein EM Hussein AM Eida MM Atwa MM Pathophysiological variability of different genotypes of human *Blastocystis hominis* egyptian isolates in experimentally infected rats Parasitol Res 2008 14 853 860 10.1007/s00436-007-0833-z 18193282

[B22] Tan KSW Mirza H Teo JDW Wu B MacAry PA Current views on the clinical relevance of *Blastocystis* spp Curr Infect Dis Rep 2010 14 28 35 10.1007/s11908-009-0073-8 21308496

[B23] Denoeud F Roussel M Noël B Wawrzyniak I Da Silva C Diogon M Viscogliosi E Brochier-Armamet C Couloux A Poulain J Segurans B Anthouard V Texier C Blot N Poirier P Ng GC Tan KSW Antiguenave F Jaillon O Aury J Delbac F Wincker P Vivarès CP El Alaoui H Genome sequence of the stramenopile *Blastocystis*, a human anaerobic parasite Genome Biol 2011 14 R29 10.1186/gb-2011-12-3-r29 21439036 PMC3129679

[B24] Clark CG van der Giezen M Alfellani MA Stensvold CR Recent developments in *Blastocystis* research Adv Parasitol 2013 14 1 32 23548084 10.1016/B978-0-12-407706-5.00001-0

[B25] Poirier P Wawrzyniak I Vivares CP Delbac F El AH New insights into *Blastocystis* spp.: a potential link with irritable bowel syndrome PLoS Pathog 2012 14 e1002545 10.1371/journal.ppat.1002545 22438803 PMC3305450

[B26] Mirza H Wu Z Teo JDW Tan KSW Statin pleiotropy prevents rho kinase-mediated intestinal epithelial barrier compromise induced by *Blastocystis* cysteine proteases Cell Microbiol 2012 14 1474 1484 10.1111/j.1462-5822.2012.01814.x 22587300

[B27] Wawrzyniak I Texier C Poirier P Viscogliosi E Tan KS Delbac F El Alaoui H Characterization of two cysteine proteases secreted by *Blastocystis* ST7, a human intestinal parasite Parasitol Int 2012 14 437 442 10.1016/j.parint.2012.02.007 22402106

[B28] Boorom KF Smith H Nimri L Viscogliosi E Spanakos G Parkar U Li LH Zhou XN Ok UZ Leelayoova S Jones MS Oh my aching gut: irritable bowel syndrome, *Blastocystis*, and asymptomatic infection Parasit Vectors 2008 14 40 10.1186/1756-3305-1-40 18937874 PMC2627840

[B29] Vogelberg C Stensvold CR Monecke S Ditzen A Stopsack K Heinrich-Grafe U Pohlmann C *Blastocystis* sp. subtype 2 detection during recurrence of gastrointestinal and urticarial symptoms Parasitol Int 2010 14 469 471 10.1016/j.parint.2010.03.009 20363362

[B30] Verma R Delfanian K *Blastocystis hominis* associated acute urticaria Am J Med Sci 2013 14 80 81 10.1097/MAJ.0b013e3182801478 23360793

[B31] Tan TC Ong SC Suresh KG Genetic variability of *Blastocystis* sp. isolates obtained from cancer and HIV/AIDS patients Parasitol Res 2009 14 1283 1286 10.1007/s00436-009-1551-5 19603182

[B32] Batista MW Pierrotti LC Abdala E Clemente WT Girao ES Rosa DRT Ianhez LE Bonazzi PR Lima AS Fernandes PFCBC Padua-Neto MV Bacchella T Oliveira APP Viana CFG Ferreira MS Shikanai-Yasuda MA Endemic and opportunistic infections in Brazilian solid organ transplant recipients Trop Med Int Health 2011 14 1134 1142 10.1111/j.1365-3156.2011.02816.x 21692958

[B33] Stensvold CR Christiansen DB Olsen KEP Nielsen HV *Blastocystis* sp. subtype 4 is common in Danish *Blastocystis*-positive patients presenting with acute diarrhea Am J Trop Med Hyg 2011 14 883 885 10.4269/ajtmh.2011.11-0005 21633023 PMC3110361

[B34] Forsell J Granlund M Stensvold CR Clark CG Evengard B Subtype analysis of *Blastocystis* isolates in Swedish patients Eur J Clin Microbiol Infect Dis 2012 14 1689 1696 10.1007/s10096-011-1416-6 22350386

[B35] Moosavi A Haghighi A Nazemalhosseini Mojarad E Zayeri F Alebouyeh M Khazan H Kazemi B Zali MR Genetic variability of *Blastocystis* sp. isolated from symptomatic and asymptomatic individuals in Iran Parasitol Res 2012 14 2311 2315 10.1007/s00436-012-3085-5 22948205

[B36] El Safadi D Meloni D Poirier P Osman M Cian A Gaayeb L Wawrzyniak I Delbac F El Alaoui H Delhaes L Dei-Cas E Mallat H Dabboussi F Hamze M Viscogliosi E Molecular epidemiology of *Blastocystis* in Lebanon and correlation between subtype 1 and gastrointestinal symptoms Am J Trop Med Hyg 2013 14 1203 1206 10.4269/ajtmh.12-0777 23458955 PMC3752823

[B37] Souppart L Moussa H Cian A Sanciu G Poirier P El Alaoui H Delbac F Boorom K Delhaes L Dei-Cas E Viscogliosi E Subtype analysis of *Blastocystis* isolates from symptomatic patients in Egypt Parasitol Res 2010 14 505 511 10.1007/s00436-009-1693-5 19953268

[B38] Fouad SA Basyoni MM Fahmy RA Kobaisi MH The pathogenic role of different *Blastocystis hominis* genotypes isolated from patients with irritable bowel syndrome Arab J Gastroenterol 2011 14 194 200 10.1016/j.ajg.2011.11.005 22305500

[B39] Petrasova J Uzlikova M Kostka M Petrzelkova KJ Huffman MA Modry D Diversity and host specificity of *Blastocystis* in syntopic primates on Rubondo Island, Tanzania Int J Parasitol 2011 14 1113 1120 10.1016/j.ijpara.2011.06.010 21854778

[B40] Scicluna SM Tawari B Clark CG DNA barcoding of *Blastocystis* Protist 2006 14 77 85 10.1016/j.protis.2005.12.001 16431158

[B41] Souppart L Sanciu G Cian A Wawrzyniak I Delbac F Capron M Dei-Cas E Boorom K Delhaes L Viscogliosi E Molecular epidemiology of human *Blastocystis* isolates in France Parasitol Res 2009 14 413 421 10.1007/s00436-009-1398-9 19290540

[B42] Stensvold CR *Blastocystis*: genetic diversity and molecular methods for diagnosis and epidemiology Trop Parasitol 2013 14 26 34 10.4103/2229-5070.113896 23961438 PMC3745667

[B43] Stensvold CR Comparison of sequencing (barcode region) and sequence-tagged-site PCR for *Blastocystis* subtyping J Clin Microbiol 2013 14 190 194 10.1128/JCM.02541-12 23115257 PMC3536234

[B44] Meloni D Poirier P Mantini C Noël C Gantois N Wawrzyniak I Delbac F Chabé M Delhaes L Dei-Cas E Fiori PL El Alaoui H Viscogliosi E Mixed human intra- and inter-subtype infections with the parasite *Blastocystis* sp Parasitol Int 2012 14 719 722 10.1016/j.parint.2012.05.012 22659011

[B45] Velasco J Gonzalez F Diaz T Pena-Guillen J Araque M Profiles of enteropathogens in asymptomatic children from indigenous communities of Merida, Venezuela J Infect Dev Ctries 2011 14 276 285 10.3855/jidc.1162 21537069

[B46] Machicado JD Marcos LA Tello R Canales M Terashima A Gotuzzo E Diagnosis of soil-transmitted helminthiasis in an Amazonic community in Peru using multiple diagnostic techniques Trans R Soc Trop Med Hyg 2012 14 333 339 10.1016/j.trstmh.2012.03.004 22515992

[B47] Saksirisampant W Nuchprayoon S Wiwanitkit V Yenthakam S Ampavasiri A Intestinal parasitic infestations among children in an orphanage in Patum Thani province J Med Assoc Thai 2003 14 S263 S270 12929999

[B48] Gualdieri L Rinaldi L Petrullo L Morgoglione ME Maurelli MP Musella V Piemonte M Caravano L Coppola MG Cringoli G Intestinal parasites in immigrants in the city of Naples (southern Italy) Acta Trop 2011 14 196 201 10.1016/j.actatropica.2010.12.003 21195044

[B49] Alfellani MA Khan AH Al-Gazaoui RM Zaid MK Al-Ferjani MA Prevalence and clinical features of *Blastocystis hominis* infection among patients in Sebha, Libya Sultan Qaboos Univ Med J 2007 14 35 40 21654943 PMC3086416

[B50] Faye O Ndir O Dieng T Gaye O Bah IB Dieng Y Faye I Diallo S Cryptosporidiosis among intestinal parasitosis in Senegalese pediatric hospital patients Dakar Med 1993 14 129 132 7758369

[B51] Gaye O Ndir O Kane A Belmachi R Ndiaye M Diouf M Diedhiou M Diallo S Intestinal parasitic diseases and tinea of the scalp in Dakar school population: influence of environmental factors on the infestation level Dakar Med 1994 14 57 61 7493523

[B52] Salem G van de Velden L Laloé F Maire B Ponton A Traissac P Prost A Intestinal parasitic diseases and environment in Sahelo-Sudanese towns: the case of Pikine (Senegal) Rev Epidemiol Sante Publique 1994 14 322 333 8085049

[B53] Ndir I Gaye A Sy M Gaye O Ndir O Prevalence of intestinal parasites at the King Baudouin health center of Guediawaye (Senegal) Dakar Med 2002 14 168 171 15776669

[B54] Stensvold CR Alfellani M Clark CG Levels of genetic diversity vary dramatically between *Blastocystis* subtypes Infect Genet Evol 2012 14 263 273 10.1016/j.meegid.2011.11.002 22116021

[B55] Tian CT Tan PC Sharma R Sugnaseelan S Suresh KG Genetic diversity of caprine *Blastocystis* from Peninsular Malaysia Parasitol Res 2013 14 85 89 10.1007/s00436-012-3107-3 22961236

[B56] Stensvold CR Alfellani MA Norskov-Lauritsen S Prip K Victory EL Maddox C Nielsen HV Clark CG Subtype distribution of *Blastocystis* isolates from synanthropic and zoo animals and identification of a new subtype Int J Parasitol 2009 14 473 479 10.1016/j.ijpara.2008.07.006 18755193

[B57] Dogruman-Al F Dagci H Yoshikawa H Kurt Ö Demirel M A possible link between subtype 2 and asymptomatic infections of *Blastocystis hominis* Parasitol Res 2008 14 685 689 10.1007/s00436-008-1031-3 18523804

[B58] Özyurt M Kurt Ö Molbak K Nielsen HV Haznedaroglu T Stensvold CR Molecular epidemiology of *Blastocystis* infection in Turkey Parasitol Int 2008 14 300 306 10.1016/j.parint.2008.01.004 18337161

[B59] Li J Deng T Li X Cao G Li X Yan Y A rat model to study *Blastocystis* subtype 1 infections Parasitol Res 2013 14 3537 3541 10.1007/s00436-013-3536-7 23892480

[B60] Eroglu F Gene A Elgun K Koltas IS Identification of *Blastocystis hominis* isolates from asymptomatic and symptomatic patients by PCR Parasitol Res 2009 14 1589 1592 10.1007/s00436-009-1595-6 19685075

[B61] Yakoob J Jafri W Beg MA Abbas Z Naz S Islam M Khan R Irritable bowel syndrome: is it associated with genotypes of *Blastocystis hominis* Parasitol Res 2010 14 1033 1038 10.1007/s00436-010-1761-x 20177906

[B62] Dominguez-Marquez MV Guna R Munoz C Gomez-Munoz MT Borras R High prevalence of subtype 4 among isolates of *Blastocystis hominis* from symptomatic patients of a health district of Valencia (Spain) Parasitol Res 2009 14 949 955 10.1007/s00436-009-1485-y 19471964

[B63] World Health Organization Microbial fact sheets World Health Organization Guidelines for Drinking Water quality (WHOGDWQ) 2011 4 Malta: Gutenberg 271 273

[B64] Leelayoova S Rangsin R Taamasri P Naaglor T Thathaisong U Mungthin M Evidence of waterborne transmission of *Blastocystis hominis* Am J Trop Med Hyg 2004 14 658 662 15211009

