# Supplementary material for: Children of Senegal River Basin show the highest prevalence of Blastocystis sp. ever observed worldwide
Source: BMC Infect Dis. 2014 Mar 25;14:164. doi: 10.1186/1471-2334-14-164 (PMC3987649; doi:10.1186/1471-2334-14-164)
Supplement: Additional file 1 — Standardized questionnaire completed for each child in the study. [file 1471-2334-14-164-S1.pdf]

Identifiant :

___ ___ ___ ___ P3V

Questionnaire enfant
(P3V : Octobre 2011-Etude Schisto-VAN)

Village ☐A ☐F☐ N Date ____/oct/2011 Heure :…………………… Enquêteur…………………… T.ext ……..ºC

Prénom ……………………………………… Nom………………………………………………… ☐ Fille ☐ Garçon

Père…………………………………………………… Mère ……………………………………………… Ecole, Classe :…………………………

Date de Naissance ……./…../…….. Âge……………………… Température auriculaire _ _ , _ ºC

Qui a répondu ? ……………………………. Lien à l'enfant …………………………… Nº concession ………….. Chef……………………………

Liens de parenté avec d'autres enfants de l'étude ? ……………………………………………………………………………………………

Partage l'habitation avec certains de ces enfants ? ☐oui ☐non lesquels ? ……………………………………………………

	Oui	Non	Quand	Durée	Traitement
Fièvre récente					
Paludisme récent					
Toux de plus que 3 semaines					
Diarrhée					
Vomissements					
Autres maladies					

Hématurie ? ☐ oui ☐ non Maux de ventre ? ☐ oui ☐non Douleurs à la miction ? ☐oui ☐non

Baignade régulière dans le fleuve ou canaux d'irrigation ☐oui ☐ non Fréquence ? ……………………………………………

Heure ?……………… Durée ? ………………………… Traitement au PZQ ?☐ oui ☐non Quand ? …………………………………

Remarques ? ………

Voyage ?☐ oui ☐ non Où ? ……………………………………. Durée ? …………….. Il y a combien de temps ?…………………

Observations relatives aux **urines** : ……………………………………………………………………………………………

Observations relatives aux **selles**: ……………………………………………………………………………………………

Taux d'hémoglobine : ☐ g/dl Taille 1 : ………. (cm) Taille 2 : ………. (cm) Poids: ………. (Kg)

Carnet de vaccination :☐ oui ☐ oui mais manquant ☐non Complètement vacciné ☐ oui ☐non

BCG …………………………. Penta 1 …………………………. Penta 2 …………………………. Penta 3 …………………………. Var-VAA ……………………

Observations : ……

………
